# Current evidence of nutritional therapy in pancreatoduodenectomy: Systematic review of randomized controlled trials

**DOI:** 10.1002/ags3.12287

**Published:** 2019-10-10

**Authors:** Kosei Takagi, Piotr Domagala, Hermien Hartog, Casper van Eijck, Bas Groot Koerkamp

**Affiliations:** ^1^ Department of Surgery Erasmus MC University Medical Center Rotterdam Rotterdam The Netherlands; ^2^ Department of Gastroenterological Surgery Okayama University Graduate School of Medicine, Dentistry, and Pharmaceutical Sciences Okayama Japan; ^3^ Department of General and Transplantation Surgery The Medical University of Warsaw Warsaw Poland

**Keywords:** enteral nutrition, immunonutrition, nutritional intervention, pancreatoduodenectomy, synbiotics

## Abstract

**Aim:**

Evidence of nutritional therapies in pancreatoduodenectomy (PD) has been shown. However, few studies focus on the association between different nutritional therapies and outcomes. The aim of this review was to summarize the current evidence of nutritional therapies such as enteral nutrition (EN), immunonutrition, and synbiotics on postoperative outcomes after PD.

**Methods:**

A systematic literature search of Embase, Medline Ovid, and Cochrane CENTRAL was done to summarize the available evidence, including randomized controlled trials, meta‐analyses and reviews, regarding nutritional therapy in PD.

**Results:**

A total of 20 randomized controlled trials were included in this review. Safety and tolerability of EN in PD was shown. Giving postoperative EN can shorten length of stay compared to parenteral nutrition; however, the effect of EN on postoperative complications remains controversial. Postoperative EN should be given only on selective indications rather than routinely used, and preoperative EN is indicated only in patients with severe malnutrition. Giving preoperative immunonutrition is considered to reduce the incidence of infectious complications; however, evidence level is moderate and recommendation grade is weak. The beneficial effect of perioperative synbiotics on postoperative infectious complications is limited. Furthermore, the effectiveness of other nutritional supplements remains unclear.

**Conclusion:**

Recently, evidence of enhanced recovery after surgery (ERAS) in PD has been increasing. Early oral intake with systematic nutritional support is an important aspect of the ERAS concept. Future well‐designed studies should investigate the impact of systematic nutritional therapies on outcomes following PD.

## Abstract 2

This review summarized the available evidence regarding nutritional therapy in patients following PD. Systematic nutritional support is an important aspect of the ERAS concept.

## Graphical abstract

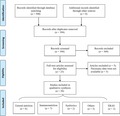

## 1 INTRODUCTION

Pancreatoduodenectomy (PD) is a highly invasive procedure in abdominal surgery. Outcomes following PD have improved due to the development of the operative technique, surgical instruments, and perioperative management; however, complication and mortality rates are still high.^1^, ^2^, ^3^ To improve clinical outcomes in gastrointestinal surgery, perioperative nutritional therapy is considered to be important.

A previous review regarding perioperative nutritional support in patients undergoing PD was published in 2006 and suggested that enteral nutrition (EN) is associated with lower incidence of postoperative infections.^4^ However, this review evaluated only four studies focused on patients who underwent PD, including two randomized controlled trials (RCT), and further studies on this issue have been reported since 2006. Furthermore, the concept of enhanced recovery after surgery (ERAS), a multimodal strategy aimed to accelerate postoperative recovery, has recently been rapidly spreading in the field of PD.^5^, ^6^

The aim of the present review was to overview the current evidence of nutritional therapies such as EN, immunonutrition (IM), synbiotics and other nutritional supplements as a nutritional aspect of the ERAS concept, and to evaluate the association between nutritional therapies and postoperative outcomes in patients undergoing PD.

## 2 MATERIALS AND METHODS

A systematic literature search of Embase, Medline Ovid, and Cochrane CENTRAL was carried out on January 11, 2019 using the following key words: diet therapy, enteral nutrition, synbiotics, supplements, enhanced recovery after surgery, and pancreatoduodenectomy (Table S1). The search was limited to RCT, meta‐analyses, and reviews in English. The present study included articles reporting outcomes of nutritional therapies in patients following PD. After removing duplicate records, abstracts were screened independently by two investigators to determine eligible studies for further analysis. Full‐text articles of the remaining records were subsequently retrieved and screened independently by two investigators. The present study is reported according to the Preferred Reporting Items for Systematic Reviewers and Meta‐Analyses (PRISMA) guidelines.^7^

## 3 RESULTS

**Figure 1:**
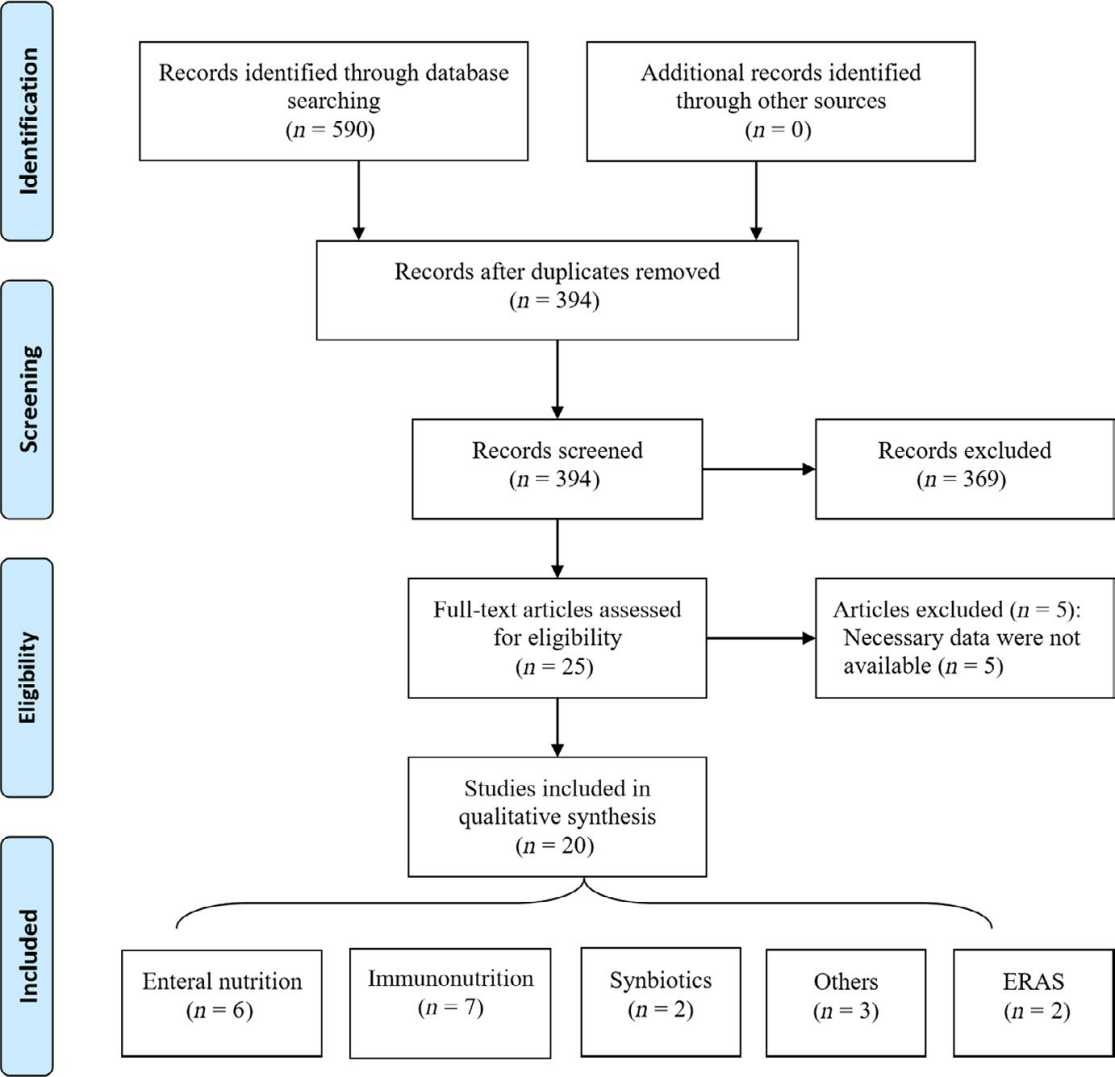
PRISMA 2009 flow diagram of articles included in the present review

A systematic search of the literature identified 590 articles, 20 RCT which matched the inclusion criteria: EN (n = 6); IM (n = 7); synbiotics (n = 2); other nutritional supplements (n = 3); and ERAS (n = 2) (Figure 1). Summary of all the included studies is represented in Table 1, showing sample size, type of intervention, timing of intervention, and outcomes.

**Table 1:** Summary of randomized controlled trials included in the present review

Study	Year	Study design	Sample size	Intervention (Timing)	Outcomes	Conclusions
Enteral nutrition						
Mack et al^8^	2004	Single center	20 vs 16	EN (Post) vs SC (Post)	Major complications: 5 vs 25% (*P *=* *.15)	Gastric decompression and EN through a double‐lumen gastrojejunostomy tube improved outcomes
					DGE: 0 vs 25% (*P *=* *.03)	
					LOS: 11.5 vs 15.8 d (*P *=* *.01)	
Grizas et al^9^	2008	Single center	30 vs 30	EN (Post) vs SC (Post)	Complications: 23.3 vs 53.3% (*P *=* *.03)	EN helped to decrease the incidence of infectious complications
					Infectious complications: 16.7 vs 46.7% (*P *=* *.025)	
					Mortality: 0 vs 6.7% (*P *=* *.49)	
Tien et al^10^	2009	Single center	123 vs 124	EN (Post) vs TPN (Post)	Major complications: 9.8 vs 9.7% (*P *=* *.98)	EN and biliopancreatic diversion minimized impacts of DGE
					Infectious complications: 21.1 vs 23.6% (*P *=* *.67)	
					DGE: 16.3 vs 21.7% (*P *=* *.27)	
					POPF: 4.9 vs 5.6% (*P *=* *.79)	
					Mortality: 1.6 vs 1.6% (*P *=* *.99)	
Liu et al^11^	2011	Single center	30 vs 30	EN (Post) vs TPN (Post)	DGE: 0 vs 20% (*P *=* *.039)	EN was superior to TPN
					POPF: 3.6 vs 26.7% (*P *=* *.039)	
					LOS: 17.8 vs 19.2 d (*P *=* *.375)	
					Mortality: 0 vs 0%	
Park et al^12^	2012	Single center	20 vs 20	EN (Post) vs TPN (Post)	DGE: 11 vs 5% (*P *=* *.485)	EN was associated with preservation of weight compared with TPN and with recovery of digestive function after PD
					POPF: 11 vs 5% (*P *=* *.485)	
					LOS: 23.2 vs 25.3 d (*P *=* *.991)	
Perinel et al^13^	2016	Multicenter	103 vs 101	EN (Post) vs TPN (Post)	Complications: 77.5 vs 64.4% (*P *=* *.04)	EN is not recommended in terms of safety and feasibility
					Infectious complications: 39.2 vs 41.6% (*P *=* *.71)	
					DGE: 34.3 vs 27.3% (*P *=* *.281)	
					POPF: 48.1 vs 27.7% (*P *=* *.012)	
					LOS: 25.8 vs 23.6 d (*P *=* *.181)	
					90‐day mortality: 9.7 vs 3% (*P *=* *.049)	
Immunonutrition						
Di Carlo et al^14^	1999	Single center	33 vs 35 vs 32	IM (Post) vs EN (Post) vs TPN (Post)	Complications: 33.5 vs 40.0 vs 59.5% (*P *=* *.05)	IM seemed to improve outcome
					Infectious complications: 9.1 vs 17.2 vs 25.0%	
					DGE: 9.1 vs 5.7 vs 12.5%	
					POPF: 9.1 vs 11.4 vs 12.4%	
					LOS: 16.3 vs 17.8 vs 19.3 d (*P *<* *.05)	
					Mortality: 3.3 vs 0 vs 6.2%	
Gianotti et al^15^	2000	Single center	71 vs 73 vs 68	EN/IM (Post) vs EN (Post) vs TPN (Post)	Complications: 33.8 vs 43.8 vs 58.8% (*P *=* *.005)	IM ameliorated the immunometabolic response and improves outcome compared to parenteral feeding
					Infectious complications: 8.4 vs 15.1 vs 22.1% (*P *=* *.04)	
					DGE: 11.3 vs 12.3 vs 14.7%	
					POPF: 9.9 vs 12.3 vs 11.8%	
					LOS: 15.1 vs 17.0 vs 18.8 d (*P *<* *.05)	
					Mortality: 2.8 vs 1.4 vs 5.8%	
Hamza et al^16^	2015	Single center	17 vs 20	IM (Peri) vs SC (Post)	Patients with IM had significantly higher total lymphocyte count on POD 3 and significantly greater rise in CD4/CD8 ratio from POD 3 to POD 7	Perioperative IM was associated with favorable modulation of the inflammatory response and enhancement of systemic immunity
Suzuki et al^17^	2010	Single center	10 vs 10 vs 10	IM (Peri) vs IM (Post) vs TPN (Post)	Infectious complications: 10 vs 60 vs 60% (*P *<* *.05)	Perioperative IM reduced stress‐induced immunosuppression
Aida et al^18^	2014	Single center	25 vs 25	IM (Pre)/EN (Post) vs EN (Post)	Infectious complications: 28 vs 60% (*P *=* *.023)	Preoperative IM may stop aggravation of complications
					DGE: 20 vs 12% (*P *=* *.44)	
					POPF: 20 vs 28% (*P *=* *.51)	
Miyauchi et al^19^	2019	Single center	30 vs 30	IM (Peri) vs IM (Pre)	Infectious complications: 13.3 vs 26.7% (*P *=* *.166)	No additional effects of perioperative IM on postoperative immunity and infectious complications compared with preoperative IM
					DGE: 6.7 vs 13.3% (*P *=* *.389)	
					POPF: 20 vs 33.3% (*P *=* *.371)	
					Mortality: 0 vs 0%	
Ashida et al^20^	2018	Single center double‐blinded	11 vs 9	IM (Pre) vs no‐IM (Pre)	Complications: 91 vs 78% (*P *=* *.57)	Preoperative IM had no marked impact on rates of postoperative hypercytokinemia or infectious complications
					Infectious complications: 55 vs 78% (*P *=* *.37)	
					POPF: 54.5 vs 44.4% (*P *=* *.99)	
					Mortality: 0 vs 0%	
Synbiotics						
Rayes et al^21^	2007	Single center double‐blinded	40 vs 40	SN (Peri) vs no‐SN (Peri)	Infectious complications: 12.5 vs 40% (*P *=* *.005)	SN reduced bacterial infection rates and antibiotic therapy
					DGE: 2.5 vs 10% (ns)	
					POPF: 7.5 vs 10% (ns)	
					LOS: 17 vs 22 d (ns)	
					Mortality: 2.5 vs 2.5% (ns)	
Yokoyama et al^22^	2016	Single center	22 vs 22	SN (Pre) vs no‐SN (Pre)	Major complications: 45 vs 27% (*P *=* *.21)	Preoperative SN did not affect the incidence of infectious complications
					Infectious complications: 41 vs 36% (*P *=* *.757)	
					DGE: 9 vs 27% (*P *=* *.12)	
					POPF: 36 vs 14% (*P *=* *.08)	
					LOS: 37 vs 35 d (*P *=* *.68)	
					Mortality: 5 vs 0%	
Other nutritional supplementation						
Jo et al^23^	2006	Single center double‐blinded	32 vs 28	Glu (Peri) vs no‐Glu (Peri)	Complications: 37.5 vs 28.6% (*P *=* *.46)	No beneficial effect of Glu supplementation on outcomes
					DGE: 12.5 vs 14.3% (ns)	
					POPF: 6.3 vs 0% (ns)	
					LOS: 14.0 vs 14.5 d (*P *=* *.20)	
					Mortality: 3.1 vs 0% (*P *=* *.35)	
Braga et al^24^	2012	Single center double‐blinded	18 vs 18	pONS (Peri) vs no‐pONS (Peri)	Plasma levels of vitamin C (*P *=* *.001), selenium (*P *=* *.07), and zinc (*P *=* *.06) were higher in the pONS group on POD 1	Perioperative pONS positively affected plasma vitamin C levels and improved total endogenous antioxidant capacity, but did not reduce oxidative stress and systemic inflammation markers
					No difference was found in C‐reactive protein levels after surgery in either group	
Zhu et al^25^	2013	Single center	38 vs 38	PUFA (Post) vs no‐PUFA (Post)	Infectious complications: 36.8 vs 57.9% (*P *<* *.05)	PUFA can improve nutritional status, decrease the incidence of infectious complications, and shorten LOS
					DGE: 7.9 vs 5.3% (ns)	
					POPF: 2.6 vs 2.6% (ns)	
					LOS: 13.5 vs 15.3 d (*P *<* *.05)	
					Mortality: 0 vs 0%	
Enhanced recovery after surgery						
Takagi et al^26^	2018	Single center	37 vs 37	ERAS (Peri) vs SC (Peri)	Complications: 32.4 vs 56.8% (*P *=* *.034)	ERAS contributed to earlier recovery and shorter hospital stay without compromising surgical outcomes
					Infectious complications: 19 vs 41% (*P *=* *.04)	
					DGE: 10.8 vs 8.1% (*P *=* *.34)	
					POPF: 18.9 vs 27.0% (*P *=* *.12)	
					LOS: 20.1 vs 26.9 d (*P *<* *.001)	
					Mortality: 0 vs 0%	
Deng et al^27^	2017	Single center	76 vs 83	ERAS (Peri) vs SC (Peri)	DGE: 20 vs 39% (*P *=* *.02)	ERAS protocol significantly relieved physiological stress and accelerated recovery thereby reducing LOS
					POPF: 51 vs 43% (*P *=* *.52)	
					LOS: 15 vs 19 d (*P *=* *.024)	
					Mortality: 0 vs 0%	

Abbreviations: DGE, delayed gastric emptying; EN, enteral nutrition; ERAS, enhanced recovery after surgery; Glu, glutamine; IM, immunonutrition; LOS, length of stay; ns, not significant; PD, pancreatoduodenectomy; pONS, preconditioning oral nutritional supplement; POPF, postoperative pancreatic fistula; PUFA, polyunsaturated fatty acids; SC, standard care; SN, synbiotics; TPN, total parenteral nutrition.

### 3.1 Enteral nutrition

Recent guidelines of the American Society for Parenteral and Enteral Nutrition for EN therapy recommended postoperative EN when feasible within 24 hours after surgery.^28^ Guidelines of the European Society for Parenteral and Enteral Nutrition for clinical nutrition in surgery recommended postoperative EN within 24 hours in patients in whom early oral nutrition cannot be started, and in whom oral intake will be inadequate for more than 7 days.^29^ However, we need to pay attention to the risks of PD‐related complications such as postoperative pancreatic fistula (POPF) and delayed gastric emptying (DGE).

In a RCT (Mack et al^8^) evaluating the effects of inserting a double‐lumen gastrojejunostomy tube after PD, patients were randomized to EN given by gastrojejunostomy tube (n = 20) or to the routine care group (n = 16). Authors reported the benefit of gastric decompression and EN through the gastrojejunostomy tube in terms of reducing DGE (0% vs 25%, *P *=* *.03) and postoperative length of stay (LOS) (11.5 days vs 15.8 days, *P *=* *.01). However, this study focused on the effects of the gastrojejunostomy tube rather than on EN; therefore, it is difficult to interpret whether the results were due to the tube or to the effects of EN.

Another RCT (Grizas et al^9^) comparing EN (n = 30) with standard oral diet (n = 30) after PD found a reduction in postoperative infections in postoperative EN of 16.7% vs 46.7% (*P *=* *.025). Regarding DGE, no significant difference was shown between the groups (10% vs 3.3%, *P *=* *.61). Effect of EN on LOS was unclear.

Effects of EN and biliopancreatic diversion with modified Roux‐en‐Y gastrojejunostomy reconstruction after PD were investigated in a RCT (Tien et al^10^). Two hundred and forty‐seven patients were randomized to the EN group with modified Roux‐en‐Y gastrojejunostomy reconstruction (n = 123) or to the control group with total parenteral nutrition (TPN) support. Results showed no significant differences between the two groups in terms of LOS, and postoperative complications including DGE (16.3% vs 21.7%, *P *=* *.27), POPF (4.9% vs 5.6%, *P *=* *.79), infections (21.1% vs 23.6%, *P *=* *.67), and mortality (1.6% vs 1.6%, *P *=* *.99). However, it remains difficult to determine whether the results are secondary to EN or to the modified reconstruction.

Effects of EN and TPN were examined by three RCT in patients undergoing PD and different outcomes were shown.^11^, ^12^, ^13^ In a RCT (Liu et al^11^), 60 patients were randomly divided into the EN group (n = 30) and the TPN group (n = 30). EN was not associated with LOS (17.8 days vs 19.2 days, *P *=* *.375). However, EN was associated with lower incidences of DGE (0% vs 20%, *P *=* *.039) and POPF (3.6% vs 26.7%, *P *=* *.039). Another RCT (Park et al^12^) comparing the EN group (n = 20) with the TPN group (n = 20) showed that EN was not associated with LOS (23.2 days vs 25.3 days, *P *=* *.991) and postoperative complications including DGE (11% vs 5%, *P *=* *.485) and POPF (11% vs 5%, *P *=* *.485). However, EN was associated with preservation of body weight and recovery of digestive function after PD.^12^ A recent multicenter RCT (Perinel et al^13^) involving nine centers in France randomized 204 patients to EN (n = 103) or TPN (n = 101) groups. The EN group had a significantly higher incidence of overall postoperative complications than the TPN group (77.5% vs 64.4%, *P *=* *.04). In addition, EN was associated with a higher incidence of POPF (48.1% vs 27.7%, *P *=* *.012). There were no significant differences in the incidence of DGE (34.3% vs 27.3%, *P *=* *.281), infectious complications (39.2% vs 41.6%, *P *=* *.71), and LOS (25.8 days vs 23.6 days, *P *=* *.181). It was concluded that EN should not be recommended in terms of safety and feasibility.

A meta‐analysis of four RCT^8^, ^9^, ^10^, ^15^ was conducted in 2013 to evaluate the safety and effectiveness of early enteral nutrition for patients with PD, including 246 patients with early EN and 238 patients with other nutritional therapies.^30^ Results showed no significant differences in DGE (OR: 0.89, 95% CI: 0.36‐2.18, *P *=* *.79), intra‐abdominal complications (OR: 0.82, 95% CI: 0.53‐1.26, *P *=* *.37), mortality (OR: 0.43, 95% CI: 0.11‐1.62, *P *=* *.21), infection (OR: 0.55, 95% CI: 0.29‐1.07, *P *=* *.08), and LOS (mean difference −0.93, 95% CI: −6.51‐4.65, *P *=* *.74). The authors concluded that early EN is safe and tolerable in patients undergoing PD. A recent literature review regarding EN in PD has also shown that EN is safe and well tolerated, but does not have clear advantages reducing DGE, POPF, postoperative hemorrhage, infectious complications and LOS.^31^ In contrast, the latest meta‐analyses comparing EN to TPN in patients after PD have shown that EN is associated with a significantly shorter LOS. However, EN had no effect on reducing postoperative complications including POPF, DGE, and infectious complications.^32^, ^33^

Guidelines for perioperative care of PD by the ERAS Society have recommended starting a normal diet without restriction after surgery, giving EN only for specific patients, and not routinely giving TPN.^5^, ^6^ Furthermore, the International Study Group on Pancreatic Surgery (ISGPS) has provided evidence on nutritional support in pancreatic surgery, encouraging early oral intake within ERAS protocols.^34^ However, they have recommended considering postoperative EN in patients who were preoperatively malnourished, those at high risk of developing malnutrition, and those who develop severe postoperative complications including those undergoing reoperation.

Regarding the timing of intervention, the effect of postoperative EN in PD has been investigated in all RCT.^8^, ^9^, ^10^, ^11^, ^12^, ^13^ Definitive advantages of preoperative EN remain unclear in patients with PD. However, even though the evidence level is low, according to the ERAS guidelines and the ISGPS consensus, preoperative nutritional support with EN, TPN, and supplements can be optimized only in severe malnourished patients.^5^, ^6^, ^34^

In summary, the safety and tolerability of EN in PD has been shown according to several meta‐analyses and a review.^30^, ^31^, ^32^, ^33^ Giving postoperative EN can shorten LOS compared to TPN; however, the effect of EN on postoperative complications following PD remains controversial. The concept of selective indication for artificial nutrition rather than routine use should be discussed.^5^, ^6^, ^15^, ^34^ Furthermore, preoperative EN should be indicated only in patients with severe malnutrition.^5^, ^6^, ^34^

### 3.2 Immunonutrition

Effect of IM has been examined over many years, and several systematic reviews have shown beneficial outcomes of IM in patients following gastrointestinal surgery.^35^, ^36^, ^37^

The earliest RCT (Di Carlo et al^14^) to address the problem of IM in patients undergoing PD was reported in 1999, comparing IM with standard enteral formula (n = 33), standard enteral formula (n = 35), and TPN (n = 32). IM included arginine, omega‐3 fatty acid and RNA, and postoperative feeding was given within 12 hours after surgery. Incidence of postoperative complications was lower in the IM group (33.5%) than in the standard group (40.0%) and the TPN group (59.5%, *P *=* *.05). In addition, the severity of infectious complications (sepsis score) was lower in the IM group than in the standard group and the TPN group (5.5 vs 7.9 vs 10.4%, *P *<* *.05), and LOS was shorter in the IM group than in the standard group and the TPN group (16.3 days vs 17.8 days vs 19.3 days, *P *<* *.05).

Another RCT (Gianotti et al^15^) comparing EN with IM group (n = 71), the EN group (n = 73), and the TPN group (n = 68) showed a lower incidence of postoperative complications in the EN with IM group than in the EN group and the TPN group (33.8% vs 43.8% vs 58.8%, *P *=* *.005), and shorter LOS in the IM group (15.1 days vs 17.0 days vs 18.8 days, *P *<* *.05).

In a RCT (Hamza et al^16^) investigating the effects of perioperative IM (n = 17) versus standard enteral nutrition (n = 20) on systematic and mucosal immunity in patients with PD, results showed that giving perioperative IM is associated with a favorable modulation of the inflammatory response and enhancement of systemic immunity. However, the effect of perioperative IM on outcomes following PD remains unclear.

The Chiba group in Japan carried out three RCT to investigate the effects of IM in patients with PD focusing on the timing of giving IM.^17^, ^18^, ^19^ The first RCT (Suzuki et al^17^) compared perioperative IM therapy (n = 10), postoperative IM therapy (n = 10), and TPN therapy (n = 10) and showed that perioperative IM reduced stress‐induced immunosuppression and the incidence of infectious complications (10% vs 60% vs 60%, *P *<* *.05). The second RCT (Aida et al^18^) compared preoperative IM therapy (n = 25) with no preoperative IM therapy (n = 25) and concluded that preoperative IM could help to establish a favorable immunonutrient profile before surgery and to protect against the aggravation of postoperative complications. The third RCT (Miyauchi et al^19^) evaluated the additional effect of perioperative IM (n = 30) compared with preoperative IM (n = 30) and showed no additional effects of perioperative IM on postoperative immunity and infectious complications compared with preoperative IM (13.3% vs 26.7%, *P *=* *.166). Accordingly, giving preoperative IM was suggested to prevent infectious complications following PD. However, the effect of IM on LOS was not investigated in these trials.

The first double‐blinded RCT (Ashida et al^20^) was conducted to compare outcomes between the control group (standard nutrition, n = 9) and the treatment group (preoperative eicosapentaenoic acid‐enriched nutrition, n = 11) after PD. There were no significant differences in perioperative interleukin‐6 levels between the two groups (*P *=* *.68). Furthermore, no significant differences were found in the incidence of infectious complications (55% vs 78%, *P *=* *.37) and overall complications (91% vs 78%, *P *=* *.57). No data on LOS were reported.

Giving preoperative IM is considered to reduce the incidence of infectious complications after PD; however, the evidence level of IM is moderate and the recommendation grade is weak according to the guidelines for perioperative care for PD by the ERAS Society.^5^, ^6^ Further well‐designed studies with a large number of patients are required to address the current evidence.

### 3.3 Synbiotics

Synbiotics consists of probiotics and prebiotics. Probiotics, live beneficial bacteria, can influence pathogenic mechanisms of bacterial translocation by increasing intestinal motility, stabilizing the intestinal barrier, and enhancing the innate immune system.^21^ Prebiotics, such as fiber, is a non‐digestible dietary ingredient that serves as a nutritional source for probiotics. Giving synbiotics may be helpful in preventing bacterial translocation especially following highly invasive surgery.

A first double‐blind RCT (Rayes et al^21^) was conducted in patients following PD, in which 40 patients received a composition of *Lactobacillus* and fiber and 40 patients received placebo (fiber only) starting the day before surgery and continuing for 8 days. The synbiotics group had a significantly lower incidence of postoperative infections than the placebo group (12.5% vs 40%, *P *=* *.005), and a shorter duration of antibiotic therapy (2 ± 5 days vs 10 ± 14 days, *P *=* *.015). However, no significant differences were found in LOS (17 days vs 22 days), other complications (23% vs 25%), and mortality (2.5% vs 2.5%).

Another RCT (Yokoyama et al,^22^) compared preoperative synbiotics (n = 22) with no synbiotics (n = 22) and showed no significant differences with respect to the incidence of infectious complications following PD (41% vs 36%, *P *=* *.76), POPF (≥grade B) (36% vs 14%, *P *=* *.08), DGE (≥grade B) (9% vs 27%, *P *=* *.12), and median LOS (37 days vs 35 days, *P *=* *.68).

Beneficial outcomes have been shown in a recent systematic review of 11 RCT in patients following highly invasive abdominal surgery.^38^ It was concluded that improving the intestinal microenvironment and intestinal barrier function before surgery is crucial to prevent postoperative infections following highly invasive surgery. Use of preoperative synbiotics could be helpful to improve intestinal microflora, and prevent bacterial translocation and the incidence of infectious complications.

Another systematic review of 28 RCT including 2511 patients following gastrointestinal surgery showed that giving perioperative synbiotics may prevent postoperative infections; however, the results need to be interpreted with caution as a result of the risk of bias and the potential publication bias.^39^

Although recent reviews has shown the effectiveness of synbiotics in preventing postoperative infections in gastrointestinal surgery,^38^, ^39^ different outcomes were shown in two RCT after PD.^21^, ^22^ Sample sizes of these trials were small, and postoperative infectious complications after PD were mainly associated with the incidence of POPF. Therefore, the effect of synbiotics may be limited especially in patients following PD.

### 3.4 Other nutritional supplements

To assess the effect of glutamine supplementation in patients undergoing PD, a double‐blinded RCT (Jo et al^23^) was conducted and the results were reported in 2006. From the second preoperative day to the fifth postoperative day, isonitrogenous amino acid with glutamine (0.2 g/kg per day) was given to 32 patients (the glutamine group), while another 28 patients received isonitrogenous amino acid (control group). Median LOS and postoperative nutritional status were not different between the two groups. In addition, no significant differences were found in overall complications (glutamine group 37.5% vs control group 28.6%, *P *=* *.46) and PD‐related complications (25.0% vs 14.3%, *P *=* *.30). This study showed no beneficial effect of glutamine supplementation in patients with PD.

Another double‐blinded RCT (Braga et al^24^) was carried out to evaluate the impact of a carbohydrate‐containing preconditioning oral nutritional supplement (pONS) enriched with glutamine, antioxidants, and green tea extract on postoperative oxidative stress. Patients were randomized to receive either pONS (n = 18) or placebo (n = 18) twice the day before surgery and once 3 hours before surgery. Giving perioperative pONS positively affected plasma vitamin C levels and improved total endogenous antioxidant capacity shortly after PD, but did not reduce oxidative stress and systemic inflammation markers.

Effect of parenteral fish oil lipid emulsion in TPN combined with EN support was evaluated in a RCT (Zhu et al^25^), including a polyunsaturated fatty acid (PUFA) group with parenteral fish oil lipid emulsion in PN combined with EN support for 5 days after PD (n = 38) and a control group (n = 38). Incidence of infectious complications in the PUFA group was significantly decreased (36.8% vs 57.9%, *P *<* *.05), as well as mean LOS (13.5 ± 3.8 days vs 15.3 ± 4.3 days, *P *<* *.05).

No significant beneficial effects of glutamine supplementation and pONS were shown in double‐blinded RCT.^23^, ^24^ In contrast, the beneficial effect of PUFA was shown in a RCT.^25^ However, this trial was not double‐blinded and the number of included patients was small. Therefore, further studies, including a large multicenter trial, are warranted for determining the effects of these nutritional supplements in patients undergoing PD.

### 3.5 Enhanced recovery after surgery

Guidelines for perioperative care for PD have been published in 2012 by the ERAS Society in which available evidence was summarized and recommended for 27 care items.^5^, ^6^ However, the evidence of ERAS pathways for PD is limited because no RCT has been conducted to examine the effect of ERAS protocols in patients with PD.

In 2013, Coolsen et al^40^ published a systematic review and meta‐analysis regarding ERAS in patients with pancreatic surgery. The authors examined eight studies, including five case‐control studies, two retrospective studies, and one prospective study, and meta‐analysis of four studies focusing on PD showed a significant difference in complication rates in favor of the ERAS group (absolute risk difference 8.2%, 95% CI: 2.0‐14.4, *P *=* *.008). They implied that ERAS protocols in pancreatic surgery helped to shorten LOS without compromising morbidity and mortality.

A further systematic review and meta‐analysis was published in 2016^41^ investigating the effects of implementing ERAS protocols following PD. In this study, 14 case‐control studies with 1409 ERAS patients and 1310 control patients were analyzed. Meta‐analysis showed that patients in the ERAS group had shorter LOS compared with those in the control group (weighted mean differences −4.17 days, 95% CI: −5.72 to −2.61, *P *<* *.001). In addition, implementation of ERAS protocols reduced DGE (OR: 0.56, 95% CI: 0.44‐0.71, *P *<* *.001), overall morbidity (OR: 0.63, 95% CI: 0.54‐0.74, *P *<* *.001), and in‐hospital costs compared to the control group. There were no statistically significant differences in other postoperative outcomes such as POPF, mortality and readmission rate.

A review by Pecorelli et al,^42^ including 17 non‐randomized studies, showed that ERAS protocols for pancreatic surgery are safe with no difference in postoperative morbidity, leading to early discharge and no increase in hospital readmissions. Furthermore, hospital costs were reduced as a result of better organization of care and resource use. In this study, authors summarized the specific elements as ERAS protocols, and nutritional therapy including immunonutrition and early oral nutrition was suggested as one of the key elements in ERAS. However, the authors stated that the role of ERAS pathways for pancreatic surgery is still unclear as high‐quality RCT are lacking.

Takagi et al^26^ carried out a RCT to examine the efficiency of ERAS protocols in patients following PD. Mean LOS in the ERAS group was significantly shorter than that in the control group (20.1 ± 5.4 days vs 26.9 ± 13.5 days, *P *<* *.001). The ERAS group had a significantly lower percentage of postoperative complications (32.4% vs 56.8%, *P *=* *.034) and infectious complications (19% vs 41%, *P *=* *.04). No significant differences between the groups were found in terms of POPF (≥grade B) (18.9% vs 27.0%, *P *=* *.12) and DGE (≥grade B) (10.8% vs 8.1%, *P *=* *.34). As this study included nutritional therapy such as immunonutrition, synbiotics, and EN in the ERAS protocols, nutritional therapy could contribute to earlier gastrointestinal function and accelerate postoperative recovery after PD.

In another RCT, Deng et al^27^ investigated the feasibility and safety of implementing the modified ERAS protocols based on colonic surgery in patients undergoing PD. A total of 159 patients were randomized into two groups: either ERAS (n = 76) or conventional protocol (n = 83). Authors reported that the ERAS group patients had shorter LOS (15 ± 8 vs 19 ± 10 days, *P *=* *.024) with comparable postoperative complications as follows: DGE (20% vs 39%, *P *=* *.02); POPF (51% vs 43%, *P *=* *.52); re‐laparotomy (4% vs 1%, *P *=* *.60); and mortality (0% vs 0%). Several postoperative recovery factors were greatly improved in the ERAS group, and there were no complications requiring readmission. However, nutritional therapy was not included as one element of the ERAS protocols in this study.

Accordingly, the evidence of ERAS in PD has been shown by recent meta‐analyses and RCT.^26^, ^27^, ^40^, ^41^, ^42^ Systematic nutritional support is an important element of the ERAS concept; however, not all studies have investigated the effect of nutritional therapy within ERAS pathways. In addition, which nutritional therapy should be used as perioperative systematic nutritional support in patients following PD? Future well‐designed studies should introduce nutritional therapies within ERAS protocols and investigate the impact of them on outcomes after PD.

## 4 CONCLUSIONS

The present review summarized the available evidence regarding nutritional therapy in patients following PD. Regarding the administration of EN, safety and tolerability of EN in PD has been shown. Giving postoperative EN compared to TPN can shorten length of hospital stay; however, the effect of EN on postoperative complications following PD remains controversial. Postoperative EN should be given only on selected indications rather than routinely used, and preoperative EN should be given only in patients with severe malnutrition. Giving preoperative IM should be considered in order to reduce the incidence of infectious complications after PD; however, evidence level of IM is moderate and recommendation grade is weak. The beneficial effect of perioperative synbiotics on postoperative infectious complications is limited in patients following PD. Furthermore, the effectiveness of other nutritional supplementation remains unclear. Recently, evidence for ERAS in PD has been increasing. Early oral intake should be allowed without restriction. In addition, systematic nutritional support should be an important aspect of the ERAS concept. Future well‐designed studies should investigate the impact of systematic nutritional therapies on outcomes following PD.

## DISCLOSURE

Conflicts of Interest: Authors declare no conflicts of interest for this article.

Ethical Approval: No ethical approval or informed consent statement was required for this review article.
