# Supplementary material for: Current evidence of nutritional therapy in pancreatoduodenectomy: Systematic review of randomized controlled trials
Source: Ann Gastroenterol Surg. 2019 Oct 10;3(6):620–9. doi: 10.1002/ags3.12287 (PMC6875945; doi:10.1002/ags3.12287)
Supplement: Supplementary file 1 [file AGS3-3-620-s001.docx]

**Supplementary Table 1.** Search strings and terms

| **Database** | **Search query** | **Number of found records** | **Number of found records without duplication** |
| --- | --- | --- | --- |
| embase.com | ('diet therapy'/exp OR immunonutrition/de OR 'enteric feeding'/exp OR 'synbiotic agent'/exp OR 'enhanced recovery after surgery'/de OR 'Enhanced Recovery After Surgery Protocol'/de OR supplementation/exp OR 'fast track surgery'/de OR (((diet* OR nutrition*) NEAR/3 (treat* OR therap* OR support* OR preoperat* OR perioperat* OR postoperat* OR pre-operat* OR peri-operat* OR post-operat* OR presurg* OR perisurg* OR postsurg* OR pre-surg* OR peri-surg* OR post-surg*)) OR immunonutrition* OR immune*-nutrition* OR ((enter* OR immune*) NEAR/6 (nutrition* OR feeding*)) OR synbiotic* OR supplement* OR vitamin* OR 'enhanced recovery after surgery' OR eras OR (diet* NEAR/3 (ketogen* OR carbohydrat*)) OR fast-track):ab,ti) AND ('pancreaticoduodenectomy'/exp OR (pancreaticoduodenectom* OR pancreatoduodenectom* OR (pancreatoduoden* NEAR/3 resect*) OR duodenopancreatectom* OR duodenohemipancreatectom* OR duoden*-pancreatectom* OR duoden*-hemipancreatectom* OR whipple OR pancreat*-duodenectom* OR hemipancreat*-duodenectom* OR ((pancreat* OR hemipancreat*) NEAR/6 duoden* NEAR/6 resect*)):ab,ti) AND ('Controlled clinical trial'/exp OR 'Crossover procedure'/de OR 'Double-blind procedure'/de OR 'Single-blind procedure'/de OR (random* OR factorial* OR crossover* OR (cross NEXT/1 over*) OR placebo* OR ((doubl* OR singl*) NEXT/1 blind*) OR assign* OR allocat* OR volunteer* OR trial OR groups):ab,ti) NOT ([animals]/lim NOT [humans]/lim) | 354 | 346 |
| Medline Ovid | (exp Diet Therapy/ OR Enteral Nutrition/ OR Synbiotics/ OR Dietary Supplements/ OR (((diet* OR nutrition*) ADJ3 (treat* OR therap* OR support* OR preoperat* OR perioperat* OR postoperat* OR pre-operat* OR peri-operat* OR post-operat* OR presurg* OR perisurg* OR postsurg* OR pre-surg* OR peri-surg* OR post-surg*)) OR immunonutrition* OR immune*-nutrition* OR ((enter* OR immune*) ADJ6 (nutrition* OR feeding*)) OR synbiotic* OR supplement* OR vitamin* OR enhanced recovery after surgery OR eras OR (diet* ADJ3 (ketogen* OR carbohydrat*)) OR fast-track).ab,ti.) AND (Pancreaticoduodenectomy/ OR (pancreaticoduodenectom* OR pancreatoduodenectom* OR (pancreatoduoden* ADJ3 resect*) OR duodenopancreatectom* OR duodenohemipancreatectom* OR duoden*-pancreatectom* OR duoden*-hemipancreatectom* OR whipple OR pancreat*-duodenectom* OR hemipancreat*-duodenectom* OR ((pancreat* OR hemipancreat*) ADJ6 duoden* ADJ6 resect*)).ab,ti.) AND (Exp Controlled clinical trial/ OR "Double-Blind Method"/ OR "Single-Blind Method"/ OR "Random Allocation"/ OR (random* OR factorial* OR crossover* OR cross over* OR placebo* OR ((doubl* OR singl*) ADJ blind*) OR assign* OR allocat* OR volunteer* OR trial OR groups).ab,ti.) NOT (Animals/ NOT Humans/) | 147 | 24 |
| Cochrane CENTRAL | ((((diet* OR nutrition*) NEAR/3 (treat* OR therap* OR support* OR preoperat* OR perioperat* OR postoperat* OR pre next operat* OR peri next operat* OR post next operat* OR presurg* OR perisurg* OR postsurg* OR pre next surg* OR peri next surg* OR post next surg*)) OR immunonutrition* OR immune* next nutrition* OR ((enter* OR immune*) NEAR/6 (nutrition* OR feeding*)) OR synbiotic* OR supplement* OR vitamin* OR 'enhanced recovery after surgery' OR eras OR (diet* NEAR/3 (ketogen* OR carbohydrat*)) OR fast next track):ab,ti) AND ((pancreaticoduodenectom* OR pancreatoduodenectom* OR (pancreatoduoden* NEAR/3 resect*) OR duodenopancreatectom* OR duodenohemipancreatectom* OR duoden* next pancreatectom* OR duoden* next hemipancreatectom* OR whipple OR pancreat* next duodenectom* OR hemipancreat* next duodenectom* OR ((pancreat* OR hemipancreat*) NEAR/6 duoden* NEAR/6 resect*)):ab,ti) | 89 | 24 |
| Total |  | **590** | **394** |
